# Is anti-hormonal treatment in DCIS of the breast a need?

**Published:** 2016-09

**Authors:** WAA Tjalma

**Affiliations:** Multidisciplinary Breast Clinic, Gynecological Oncology Unit, Department of Obstetrics and Gynecology, Antwerp University Hospital - University of Antwerp, Belgium.

**Keywords:** DCIS, tamoxifen, anastrozole, treatment, survival, morbidity

## Abstract

Ductal carcinomas in situ (DCIS) represent one fifth of all detected breast cancers. The detection of DCIS can be regarded as collateral damage of breast cancer screening. The treatment of DCIS is based on surgery with or without radiotherapy. Women treated for DCIS have a 10 years survival of 98 %. Could there be a role for systemic therapy in case of a DCIS? Recent published studies suggest there is. However, anti-hormonal therapy (tamoxifen, anastrozole) in DCIS causes an increased morbidity without a reduced mortality. There is an urgent need for evidence-based guidelines in the management of DCIS in order to make appropriate shared decisions.

About 20 % of all detected breast cancers are ductal carcinomas in situ (DCIS). “In situ” means that there is no infiltration in the basal membrane and it is therefore considered to be a precursor to invasive disease (Van Cleef et al,. 2014). Some precursor lesions have a minimal malignant potential, while others have a high invasive potential. The last thirty years the incidence and treatment of DCIS have increased, without a decline in the incidence of invasive breast cancer (Ozanne et al., 2011). This suggests overdiagnosis and overtreatment. In other words, the diagnosis of DCIS can be regarded as collateral damage of breast cancer screening. The survival of DCIS is excellent (98% 10 years survival) (Tjalma, 2003). In many institutions the treatment of DCIS is almost equal to the treatment of an invasive disease. The management of DCIS should be more individualised. A practical tool is the Van Nuys Prognostic Index (VNPI) (Asjoe et al., 2007; Van Cleef et al., 2014). The VNPI is based on the patient age, tumour size, tumour margins and pathological grade and stratifies patients into three groups. The low-risk group treated by breast conservative surgery alone, the intermediate group treated by breast conservative surgery and radiotherapy and the high-risk group treated by mastectomy. Still a large group of physicians feel that breast conservative surgery for DCIS should always be combined with radiotherapy despite the fact that there is no survival advantage. Recently the **Society of Surgical Oncology** (SSO), the **American Society for Radiation Oncology** (ASTRO) and the **American Society of Clinical Oncology** (ASCO) published together their new guideline (Morrow et al., 2016a; Morrow et al., 2016b; Morrow et al., 2016c). According to this guideline, DCIS can be treated by breast conservative surgery (BCS) if the tumour free margin is 2 mm and if radiotherapy is added. A guideline about a margin will reduce the overtreatment and consequently the morbidity because re-excision is done in about one third of the women who receive BCS for their DCIS. Nevertheless, still advising radiotherapy for all women with DCIS who have been treated by BCS is overtreatment. There is also an increasing trend in contralateral prophylactic mastectomy (CPM) for women with DCIS (Elsayegh et al., 2014). For women who tested positive for BRCA mutations this seems logical. But 25 % of women who tested negative for a BRCA mutation still elected for CPM (Elsayegh et al., 2014). The annual risk for a woman with DCIS to develop either invasive cancer or DCIS in the contralateral breast is 0.6 % (Tuttle et al., 2009). In women with DCIS not genetically tested, the CPM is also increasing. In a study of 51,030 patients with DCIS, during the period of 1998 and 2005, the CPM rate was 4.1% for all surgically treated patients and 13.5% for patients undergoing mastectomy (Tuttle et al., 2009). In 2005 the CPM rate for all surgically treated patients (including breast-conserving surgery) was 5.2 % and for all patients who underwent mastectomy to treat DCIS (excluding patients undergoing breast- conserving surgery) was 18.4 % (Tuttle et al., 2009). The 10 years survival and mortality for DCIS is respectively 98 % and 2%. A CPM is therefore not likely to give any survival advantage.

Two recently published randomized trials reported the value of anastrozole use versus tamoxifen in patients with DCIS (Forbes et al., 2016; Margolese et al., 2016). Reading these studies, one got the impression that there was a need for adjuvant hormonal therapy in DCIS. However, in the IBIS II trial there was no statistically significant difference in overall recurrence (HR 0.89 [95% CI 0.64–1.23]) between the two groups (Forbes et al., 2016). In the National Surgical Adjuvant Breast and Bowel Project (NSABP) B-35 trial there was a significant difference in the breast cancer-free interval in favour of the anastrozole group (HR 0.73 [95% CI 0.56– 0.96]) (Margolese et al., 2016). But anastrozole was only superior in postmenopausal patients younger than 60 years (Margolese et al., 2016). The morbidity should not be underestimated with more thromboembolic and uterine cancer events in the tamoxifen group and more osteoporotic fractures and myalgia in the anastrozole group.

Nevertheless, when you scrutinise the data you realise that there was no significantly difference in ipsilateral recurrence for DCIS or for the formation of ipsilateral invasive cancer. There was only a significant reduction in contralateral invasive breast cancer (Margolese et al., 2016). In the systematic review of tamoxifen versus no additional treatment in DCIS patients, there was a ant reduction in the risk of new DCIS events in both the ipsilateral (HR 0.75 [95% CI 0.61-0.92]) and contralateral (RR 0.50 [95% CI 0.28-0.87]) breast (Staley et al., 2014). There was also a statistically significant reduction in contralateral breast cancers (RR 0.57 [95% CI 0.39-0.83]), but there was no significant reduction in invasive breast cancers in the ipsilateral breast after tamoxifen use (HR 0.79 [95% CI 0.62- 1.01]) (Staley et al., 2014). The decision to give adjuvant anti-hormone therapy in DCIS is questionble as it has no effect on the mortality and it does have significantly adverse effect on the quality of life (Ganz et al., 2016). The data from the RCTs indicated that anti hormonal therapy reduced only the risk of contralateral invasive breast cancer in DCIS patients. There was no reduction in invasive breast cancer if the ipsilateral breast was treated by surgery and/or radiotherapy (Forbes et al., 2016; Margolese et al., 2016).

The use of the word recurrence in both studies is misleading. An in situ carcinoma doesn’t metastasis. There is no need to treat a non-invasive disease systemically. The administration of anti-hormonal therapy has an impact on the prevention of invasive breast cancer in high risk women in general and in women with DCIS only on the “normal” breast” or contralateral breast. Despite the impact of the anti-hormonal therapy on the prevention of breast cancer, there was no impact on the mortality. In situ carcinoma of the breast is a local disease and not a systemic disease as invasive breast cancer. Local disease should be treated by local therapy only.

DCIS creates Dilemmas, Confusions, Inconsistencies and Scariness. There is a tendency for overtreatment of these premalignant lesions. The overtreatment can be done by surgery and/or radiotherapy and/or systemic therapy. The current risk-driven approach costs a lot of money, creates morbidity without increased survival. There is an urgent need for evidence-based guidelines in the management of DCIS in order to make appropriate shared decisions.
